# Perceived Stress During Late Pregnancy and Infant Body Composition at 1 Month

**DOI:** 10.1210/jendso/bvae222

**Published:** 2025-01-28

**Authors:** Xiaoran Yang, Sandrah P Eckel, Luis E Maldonado, Tingyu Yang, Xinci Chen, Mario Vigil, Claudia M Toledo-Corral, Genevieve F Dunton, Brendan H Grubbs, Laila Al-Marayati, Deborah Lerner, Nathana Lurvey, Rima Habre, Shohreh F Farzan, Theresa M Bastain, Carrie Breton

**Affiliations:** Department of Population and Public Health Sciences, Keck School of Medicine, University of Southern California, Los Angeles, CA 90032, USA; Department of Population and Public Health Sciences, Keck School of Medicine, University of Southern California, Los Angeles, CA 90032, USA; Department of Population and Public Health Sciences, Keck School of Medicine, University of Southern California, Los Angeles, CA 90032, USA; Department of Population and Public Health Sciences, Keck School of Medicine, University of Southern California, Los Angeles, CA 90032, USA; Department of Population and Public Health Sciences, Keck School of Medicine, University of Southern California, Los Angeles, CA 90032, USA; Department of Population and Public Health Sciences, Keck School of Medicine, University of Southern California, Los Angeles, CA 90032, USA; Department of Health Sciences, California State University, Northridge, Northridge, CA 91330, USA; Department of Population and Public Health Sciences, Keck School of Medicine, University of Southern California, Los Angeles, CA 90032, USA; Department of Obstetrics and Gynecology, Keck School of Medicine, University of Southern California, Los Angeles, CA 90089, USA; Department of Obstetrics and Gynecology, Keck School of Medicine, University of Southern California, Los Angeles, CA 90089, USA; Eisner Health, Los Angeles, CA 90015, USA; Eisner Health, Los Angeles, CA 90015, USA; Department of Population and Public Health Sciences, Keck School of Medicine, University of Southern California, Los Angeles, CA 90032, USA; Department of Population and Public Health Sciences, Keck School of Medicine, University of Southern California, Los Angeles, CA 90032, USA; Department of Population and Public Health Sciences, Keck School of Medicine, University of Southern California, Los Angeles, CA 90032, USA; Department of Population and Public Health Sciences, Keck School of Medicine, University of Southern California, Los Angeles, CA 90032, USA

**Keywords:** maternal perceived stress, infant adiposity, pediatric obesity risk

## Abstract

**Context:**

Worldwide, obesity remains one of the most challenging crises with children being one of the most susceptible populations. The effect of maternal stress during pregnancy on newborn body composition, measured by fat mass and lean mass has, not been extensively studied.

**Objectives:**

We evaluated the association between perceived stress during late pregnancy and infant adiposity at 1 month and assessed effect modification by infant sex and preterm birth.

**Methods:**

Mother-infant dyads (N = 138) were included from the ongoing MADRES cohort. Maternal perceived stress during late pregnancy was measured by the 10-item Perceived Stress Scale (PSS), as a cumulative score, during the third trimester. Infant adiposity measures, collected at 1 month by EchoMRI, included weight, fat mass (FM), and lean mass with FM-related ratios derived. Multivariable linear regression models with interaction terms were performed.

**Results:**

Most mothers reported low to moderate stress (mean ± SD PSS: 13.2 ± 5.6) during late pregnancy. A 1-SD higher PSS was associated with higher FM% (FM (g)/weight (g): β = 0.78%; 95% CI, 0.13-1.44) but we did not find significant associations for the other adiposity measures. Statistically significant effects of perceived stress on FM-related measures were observed in male infants and preterm infants (both *P* for interaction <.05) but were null among female infants or term infants.

**Conclusion:**

In this predominately low-income Hispanic population, perceived stress during late pregnancy was associated with higher FM-related body composition measures during early infancy; this association was stronger among male and preterm infants compared to the overall population and other subgroups.

Obesity has remained one of the most challenging global crises since it was declared an epidemic by the World Health Organization in 1997 [1]. In the United States, obesity has resulted in an estimated $260.6 billion in aggregate obesity-related medical costs among adults from 2001 to 2016 [2]. There is growing concern for children who are more susceptible to obesity and more likely to develop severe obesity-related disorders at earlier ages [3]. As obesity is often lifelong, one of the most effective strategies for addressing the obesity epidemic is to identify the risk factors and potential causes of obesity in the early stages of life [4]. Several well-studied exposures are linked to the development of obesity, including inadequate physical activity, excessive caloric intake, and a diet rich in simple sugars and saturated fatty acids [5]. However, the effect of these obesogenic exposures only partially accounts for the obesity risk [6], due to large variations in individual response in weight gain and fat mass accumulation on exposure to the same obesogenic exposures (eg, caloric intake) [7].

A growing body of epidemiological and experimental evidence in humans and animals suggests that development of obesity begins in utero [8]. The fetus responds to adverse factors experienced from the maternal environment with structural and functional changes in the fetal system [9]. The third trimester of gestation is considered the most sensitive period for obesity risk, where formation of adipocytes from stem cell precursors primarily occurs [8]. Several studies suggest that maternal conditions during pregnancy (eg, gestational diabetes) [10], suboptimal maternal diet [11], and social and environmental stressors [12-15] contribute to obesity risk in newborns and children. For instance, maternal stress in pregnancy has been associated with higher proxy measures of adiposity—namely, weight, body mass index (BMI) with or without (w/o) z-score and fat mass, at birth and during childhood [12, 16]. Furthermore, sex-specific responses to maternal stress in growth rate during pregnancy have been also suggested by several studies, and male fetuses tend to have stronger responses compared to female fetuses [16, 17].

However, limited research has been conducted to understand the effects of maternal perceived stress on the development of obesity during infancy, and preterm infants have been excluded in most of the existing studies because of their unique physiology [18]. Meanwhile, the importance of newborn adiposity in the Hispanic population has not been extensively studied. In the United States, Hispanic women and children have higher rates of being overweight or obese compared to other race/ethnicity groups, with the exception of the Black population [19]. Hispanic women are also at higher risk of mental health disorders because of acculturation, the stigma associated with seeking help for mental health, and barriers to health care access [20]. Thus, it is important to understand the specific risk factors at early stages of life in the Hispanic population that might contribute to the obesity epidemic so that appropriate early intervention strategies can be developed.

In this study, we aimed to evaluate the association between perceived stress during late pregnancy and infant adiposity in the first month of life using quantitative magnetic resonance scans (EchoMRI Adolescent Body Composition Analyzer) to measure infant adiposity (ie, fat mass and lean mass [LM]). We hypothesized that higher perceived stress during late pregnancy is associated with increased infant adiposity, and is particularly associated with higher proportion of FM. Furthermore, we evaluated whether this association differed by infant sex and preterm birth status.

## Materials and Methods

### Study Population and Study Design

The Maternal and Developmental Risks from Environmental and Social Stressors (MADRES) pregnancy cohort study comprises 1066 women (and 914 live births to date) from urban Los Angeles recruited from November 2015 through April 2023 and for whom follow-up is ongoing. The MADRES study's methodology and protocols have been previously outlined [21]. In summary, inclusion criteria included being less than 30 weeks’ pregnant, at least 18 years of age, and speaking fluent English or Spanish. Exclusion criteria for the study included multiple gestation, the inability to participate and provide consent due to a physical, mental or cognitive disability, current incarceration, or HIV-positive status. At the beginning of the study, each participant provided informed consent, and the University of Southern California's institutional review board approved all aspects of the study.

**Figure 1.**
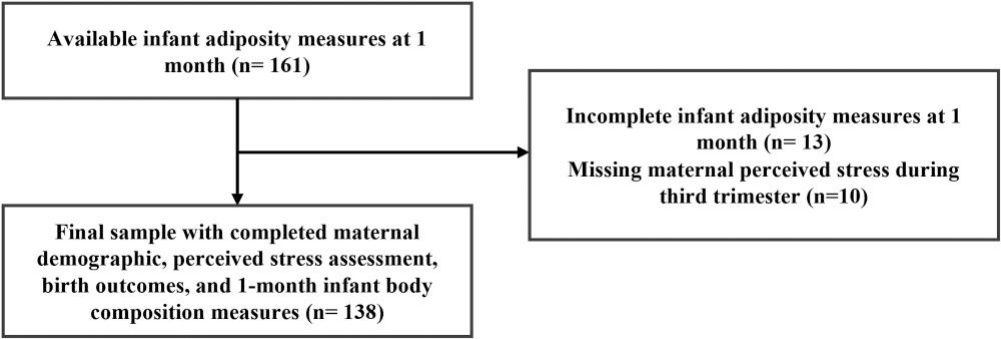
Consort diagram of included participants.

For this substudy, 161 infants had their body composition measured using the EchoMRI Adolescent Body Composition Analyzer at the 1-month study visit (age range: 31-50 days). After data processing and quality control, we excluded 13 mother-infant dyads with incomplete body composition data, and an additional 10 dyads were removed for missing maternal Perceived Stress Scale (PSS). The final analytic sample included 138 mother-infant dyads and the participant characteristics were similar to the whole MADRES cohort. The consort diagram illustrating the sample selection is shown in Fig. 1.

### Perceived Stress Scale Collection

Maternal perceived stress was measured by the 10-item PSS, which has been widely utilized and has been validated for use in Spanish-speaking populations [22, 23]. We collected the PSS once via interviewer-administered questionnaire in either Spanish or English during the first and third trimesters. The scale evaluates the level of stress perceived by the participant with questions that assess the extent to which one's life has been uncontrollable, unpredictable, and overwhelming in the past month (30 days) [23]. Responses were recorded using a Likert scale that ranges from never (a score of 0) to very often (a score of 4). The individual responses were summed to produce a total PSS score, which ranges from 0 to 40. Higher scores indicate higher levels of perceived stress. In previous research, these scores have been classified into 3 categories: low stress (0-13), moderate stress (14-26), and high stress (27-40) [24]. However, in this study, we modeled the PSS score in its continuous form because no clinically significant categories have been established.

### Infant Body Composition at 1 Month and Birthweight

Infant body composition was measured by the EchoMRI Adolescent Body Composition Analyzer, which is an instrument that utilizes quantitative magnetic resonance [25]. EchoMRI (adolescent version) can be used to assess body composition in humans up to 80 kg, and it has shown high precision and reproducibility for estimating FM, LM, and body water [26]. This system has been validated in several studies [26, 27]. All measurements were taken in triplicate, and the average of the 3 measurements was used in the analysis. Infants with any measurements flagged as unsuccessful were not included in the final sample. Outcomes measured at 1 month by EchoMRI and used in the current study comprised: (1) weight (grams [g]); (2) body length (cm); (3) FM (g); and (4) LM (g). Additionally, 3 outcomes were derived using EchoMRI measures including: (1) fat mass index (FMI) = FM/body length (kg/m^2^); (2) FM/LM ratio; (3) FM% = (FM [g]/weight[g]) × 100.

Birthweight was abstracted from electronic medical records, but, if unavailable, then birthweight was retrieved from interviewer-administered questionnaire 7 to 14 days after delivery. Missing birthweight (n = 3) was imputed with the mean birthweight in the current study.

### Covariates and Effect Modifiers

A directed acyclic graph was used to visualize covariates using Dagitty [28] and minimal sufficient adjustment sets for estimating the total effect of maternal perceived stress (measured by PSS score) exposure on birthweight and 1-month infant body composition measures consisted of Hispanic ethnicity (yes/no), level of attained education (less than high school, high school diploma or equivalent, more than high school), maternal age (continuous in years), prepregnancy BMI (continuous in kg/m^2^), infant sex (male, female), breastfeeding at first month (exclusive breastfeeding, breastfeeding and with formula, formula only), and infant age at EchoMRI measurement (continuous in days) (Supplementary Fig. S1 [29]). Other important variables were evaluated for confounding effect, but they were not included in the final model because they did not significantly change the effect estimated as described in the statistical analysis section. Most covariates were self-reported via interviewer-administered questionnaires in either English or Spanish except for prepregnancy BMI and gestational age at birth. Prepregnancy BMI in kg/m^2^ was calculated using self-reported prepregnancy weight and study measured length with a stadiometer. Variables related to study design included gestational age at birth and infant age at EchoMRI measurement. Gestational age at birth was calculated and standardized by a hierarchy of methods [30]. A first trimester (<14 weeks’ gestation) ultrasound measurement of crown-rump length was ideal if available (54%), but, if missing, a second trimester (<28 weeks’ gestation) ultrasound measurement of fetal biparietal diameter was used (22%). If no measurements from an early ultrasound were available, gestational age at birth was established from a physician's best clinical estimate from the electronic medical record (24%). Gestational age at birth was further dichotomized as preterm- (≤36 weeks) and term (>36 weeks). Infant age at EchoMRI measurement was calculated by subtracting the number of days between the infant's date of birth and the date of the EchoMRI measurement. Lastly, maternal diet in pregnancy was evaluated as a mediator in sensitivity analysis. Maternal diet during the third trimester measured in a subset of participants (n = 120) using the validated and web-based National Cancer Institute's Automated Self-Administered 24-Hour Dietary Assessment Tool (versions 2016-18) [31]. Dietary patterns were then derived. These included dietary patterns of solid fat, refined grain, and cheese score, dietary pattern of vegetables, oils, and fruit score and average total calories intake (kcal). Details of solid fat, refined grain, and cheese score and vegetables, oils, and fruit deviation were previously published [32].

### Statistical Analysis

Continuous variables including PSS, birthweight, infant body composition measures at 1 month (weight, body length, FM, LM, FMI, FM/LM ratio, and FM%), and covariates (maternal age, gestational age at birth, prepregnancy BMI, diet at trimester 3, and infant age at EchoMRI measurement) were determined using means and SDs. Frequencies and percentages were used for categorical variables (level of attained education, Hispanic ethnicity, income, infant sex, preterm birth status, breastfeeding at first month).

Linear regression models were used to evaluate the relationship between continuous PSS scores during late pregnancy, birthweight and 1-month infant body composition measures, adjusted for covariates including Hispanic ethnicity, level of attained education, maternal age, prepregnancy BMI, infant sex, breastfeeding at first month, and infant age at EchoMRI measurement. Diabetes status (ie, chronic diabetes, gestational diabetes, no diabetes), recruitment site, and room temperature during the EchoMRI scan were evaluated for potential confounding but were not included in final models because they did not change the total effect of maternal perceived stress on birthweight and 1-month infant body composition (change in effect estimates <10%). Additionally, birth weight and maternal diet at trimester 3 were not included in the model because both variables could be potential mediators [33, 34].

To determine if the associations of maternal PSS scores on birthweight and 1-month infant body composition measures were modified by infant sex and preterm birth status (cutpoint ≤36 weeks), we added an interaction term to the fully adjusted model separately for each effect modifier. The model estimates for each stratum were obtained from the model with interaction term for each effect modifier, respectively.

Several sensitivity analyses were conducted to further evaluate the robustness of results. We first examined if additional adjustment for household income, diabetes status, recruitment site, and room temperature during the EchoMRI scan influenced our results. In addition, because, as noted previously, birth weight and maternal diet during the third trimester might be potential mediators, we additionally adjusted for them in the model individually to determine if these 2 potential mediators might influence our results. We further conducted mediation analysis by running linear regression models on total, direct, and indirect effects for these 2 mediators if the estimates from the previous 2 adjusted models changes more than 10%. Last, we examined the effect of maternal stress during early pregnancy on birthweight and 1-month infant body composition measures as a sensitivity analysis (n = 97) to understand if the effect of stress remains similar or not over the pregnancy course.

Data management and linear regression models were conducted in SAS Version 9.4. All models met modeling assumptions, multicollinearity assessed from the variance inflation factor indicated no concerns (ie, variance inflation factor < 5), and all tests used 2-sided hypotheses with α = .05.

## Results

### Participant Characteristics

Demographic and participant characteristics are shown in Table 1. The participants had mean (SD) maternal age of 28.7 (5.8) years at study enrollment, with 69.6% of the population categorized as overweight or obese prior to pregnancy (mean prepregnancy BMI ± SD: 29.5 ± 7.6 kg/m^2^). The mothers were predominantly Hispanic (86.2%) and had a high school education or less at study entry (60.2%). Infant sex was relatively balanced with 54% (n = 74) female, 13% (n = 18) infants were born preterm (≤36 weeks), and 87% (n = 120) infants born at term (>36 weeks). On average, infants had a birth weight of 3380.3 (440.3) grams.

**Table 1.** Participant characteristics of mother-infant pairs (n = 138)

Variables	N (%)/Mean (SD)
**Mothers**	
** Maternal age at study entry, y**	28.7 (5.8)
** Prepregnancy BMI, kg/m^2^**	29.5 (7.6)
** Perceived Stress Scale score during trimester 3**	13.2 (5.6)
** Hispanic ethnicity**	119 (86.2%)
** Highest education level**	
** **Less than high school	36 (26.1%)
** **High school diploma or equivalent	47 (34.1%)
** **More than high school	55 (39.9%)
** Household annual income**	
** **Less than $15 000	35 (25.4%)
** **$15 000-$49 999	47 (34.1%)
** **$50 000 or more	8 (5.8%)
** **Selected “Don’t Know”	48 (34.8%)
**Infants**	
** Sex, female**	74 (53.6%)
** Birth weight, g**	3380.3 (440.3)
** Gestational age at birth, weeks**	39.0 (1.6)
** Preterm birth (≤36 weeks), yes**	18 (13.0%)
** Anthropometric and body composition measures at month 1*^a^***	
** **Weight, g	4635.2 (652.6)
** **Length, cm	54.4 (2.4)
** **Average fat mass, g	1578.0 (328.5)
** **Average lean mass, g	2952.3 (366.7)
** **FMI, kg/m^2^	5.3 (0.9)
** **FM/LM	0.5 (0.1)
** **FM%	33.8 (4.0)
** Breastfeeding at month 1**	
** **Exclusively breast feeding	45 (32.6%)
** **Breast feeding + formula	58 (42.0%)
** **Formula only	35 (25.4%)

Abbreviations: BMI, body mass index; FM, fat mass; FMI, fat mass index; LM, lean mass.

^*a*^All the measures were collected around 31-50 days after birth.

### PSS Score and Infant Body Composition Measures

The mean (SD) PSS score during late pregnancy was 13.2 (5.6) of a total possible score of 40, suggesting that most of the mothers had low to moderate levels of stress [24]. We observed a 0.78% (95% CI, 0.13-1.44; *P =* .02) higher FM% at 1 month of age associated with a 1-SD higher PSS score (5.6 points), after adjusting for covariates. We did not find significant associations with PSS score and other infant body composition measures. (Table 2).

**Table 2.** Association between maternal perceived stress during late pregnancy and infant birthweight and 1-month body composition measures per SD increase in PSS score (n = 138)

	Crude *β* (95% CI)	*P*	Adjusted *β* (95% CI)*^a^*	*P*
Birth weight, g*^b^*	51.82 (−22.32 to 125.97)	.17	54.36 (−22.26 to 130.99)	.16
Weight, g	50.11 (−60.23 to 160.46)	.37	0.27 (−98.95 to 99.49)	1.00
Length, cm	0.22 (−0.19 to 0.62)	.30	0.01 (−0.36 to 0.37)	.98
Average FM, g	47.16 (−7.98 to 102.29)	.09	32.36 (−20.63 to 85.34)	.23
Average LM, g	38.26 (−23.58 to 100.10)	.22	7.81 (−45.07 to 60.69)	.77
FMI, kg/m^2^	0.13 (−0.02 to 0.27)	.09	0.12 (−0.03 to 0.27)	.10
FM/LM	0.01 (0.00-0.02)	.13	0.01 (0.00-0.02)	.11
FM%	0.72 (0.06-1.38)	.**03***^c^*	0.78 (0.13-1.44)	.02*^c^*

Abbreviations: BMI, body mass index; FM, fat mass; FMI, fat mass index; LM, lean mass.

^*a*^All estimates adjusted for Hispanic ethnicity, prepregnancy BMI, education, infant sex, age at EchoMRI measurement, maternal age at birth, and breastfeeding status at 1 month.

^*b*^The estimates for birth weight did not adjust for breastfeeding status at month 1 and age at EchoMRI measurement.

^*c*^ *P* < .05.

**Figure 2.**
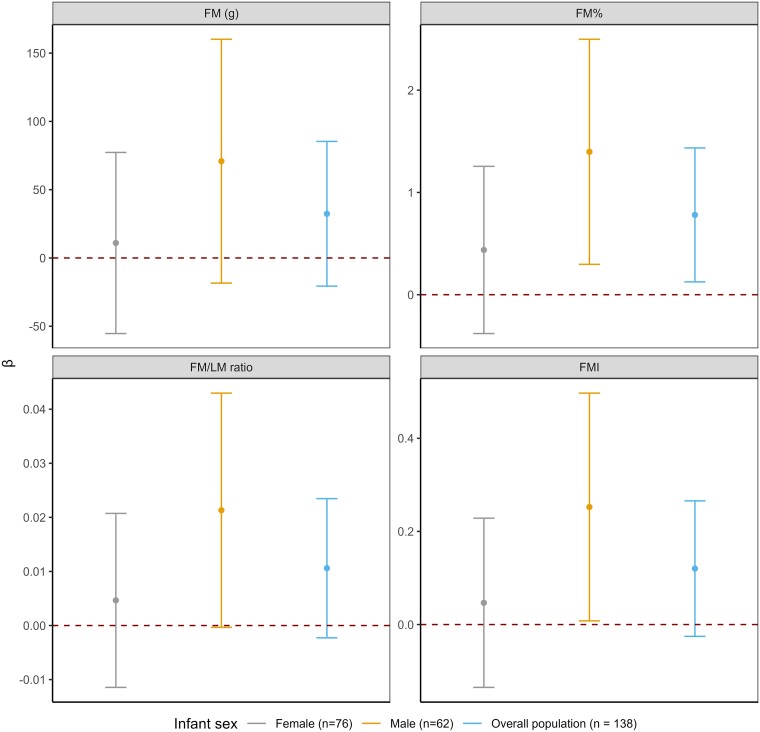
Association between maternal perceived stress during late pregnancy and fat mass-related infant body composition measures at the 1-month study visit per SD increase in PSS score, stratified by infant sex. Abbreviations: BMI, body mass index; FM, fat mass; FMI, fat mass index. Notes: (1) All estimates adjusted for Hispanic ethnicity, prepregnancy BMI, education, infant sex, age at EchoMRI measurement, maternal age at birth, and breastfeeding status at 1 month. (2) All estimates were obtained from adjusted model with interaction term because of small sample size.

We evaluated whether infant sex modified the effects of maternal perceived stress. Figure 2 presents the FM-related infant adiposity measures differences at 1 month per SD increase in PSS score during the late pregnancy, stratified by infant sex. When assessing the univariate association between PSS score and infant sex, there was not a significant association between the 2 variables (*P*  *=* .51). In the linear regression model with infant sex as interaction term, we found that the association between maternal perceived stress and FMI or FM% was only significant in male infants (per SD increase in PSS score: FMI *β* = .25 kg/m^2^; 95% CI, 0.01-0.50; *P =* .04; FM% *β* = 1.40%; 95% CI, 0.30-2.5; *P =* .01) compared to female infants (FMI *β* = .05 kg/m^2^; 95% CI, −0.14 to 0.23; *P =* .61; FM% *β* = .44%; 95% CI, −0.38 to 1.26; *P = .29*), adjusted for same set of covariates (formal tests of interaction *P* values: FMI: 0.19 and FM%: 0.17). Supplementary Table S1A [29] shows the remaining nonsignificant results for maternal perceived stress and other infant body composition measures, stratified by infant sex.

**Figure 3.**
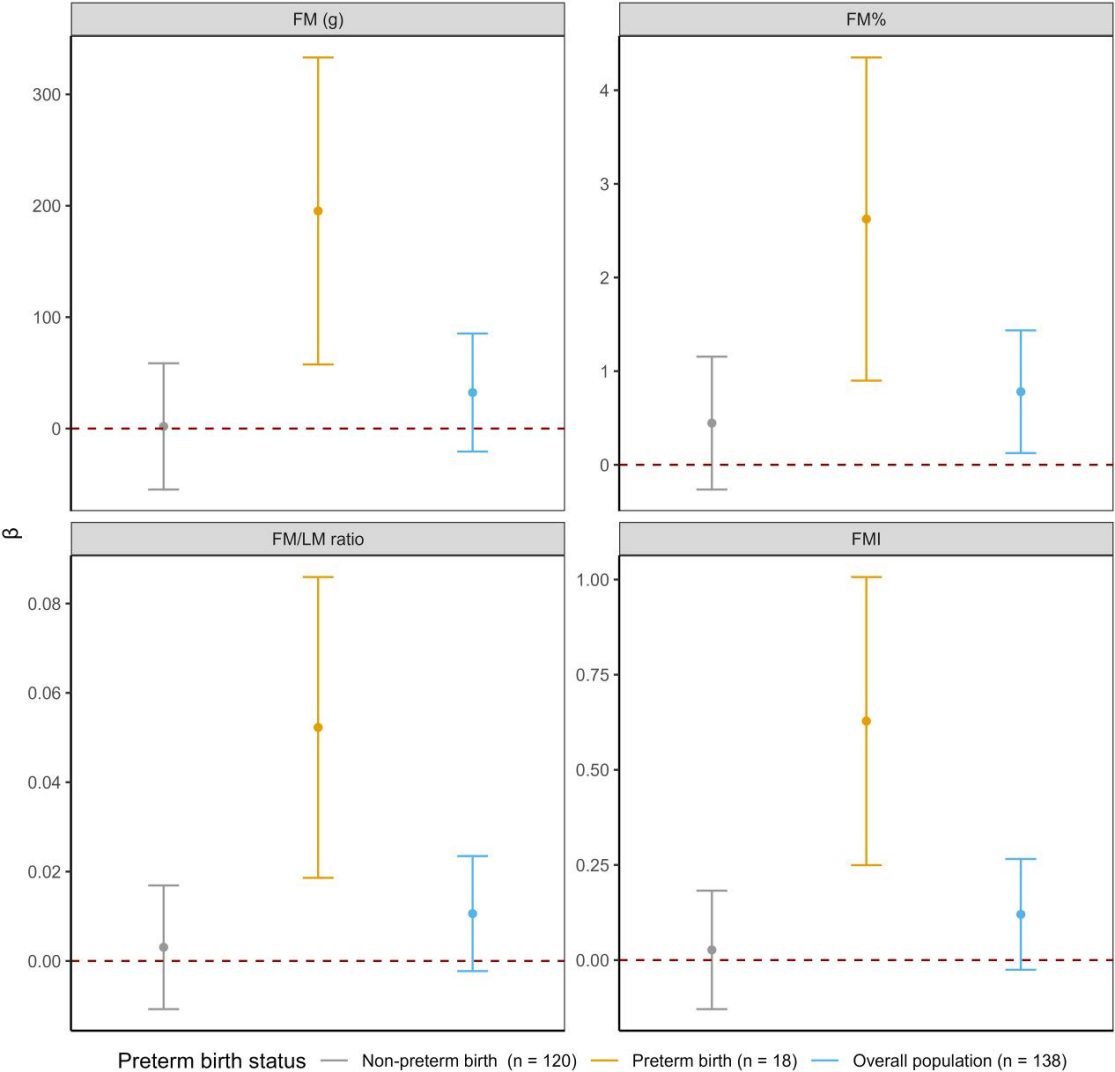
Association between maternal perceived stress during late pregnancy and weight and fat mass-related infant body composition measures at month 1 study visit per SD increase in PSS score, stratified by preterm birth status. Abbreviations: BMI, body mass index; FM, fat mass; FMI, fat mass index. Notes: (1) All estimates adjusted for Hispanic ethnicity, prepregnancy BMI, education, infant sex, age at EchoMRI measurement, maternal age at birth, and breastfeeding status at 1 month. (2) All estimates were obtained from adjusted model with interaction term because of small sample size.

Additionally, we evaluated whether preterm infants were more vulnerable to the effects of maternal perceived stress compared to term infants. The univariate association between PSS score and preterm birth status was not significant, suggesting that there is no indirect effect from PSS score and infant adiposity measures through preterm birth status (*P*  *=* .88). In the linear regression model with dichotomized preterm birth status as interaction term, we found that the association between maternal PSS scores and several infant body composition measures were stronger and only significant in infants born preterm compared to infants born full term. For example, compared to term infants, maternal perceived stress was associated with higher weight and averaged FM in infants born preterm, after adjusting covariates (preterm vs term) per SD increase in PSS score—weight: *β* = 307.69 vs −58.34 g; 95% CI, 55.90-559.48 vs −161.89 to 45.21; FM: *β* = 195.35 vs 1.93 g; 95% CI, 57.56-333.13 vs −54.74 to 58.59) (Fig. 3). The formal tests of interaction were statistically significant for multiple body composition measures with preterm birth status (*P*  *<* .05). Supplementary Table S1B [29] shows the remaining nonsignificant results for maternal perceived stress and other infant body composition measures, modified by preterm birth status.

Last, we assessed the robustness of the results of maternal perceived stress on infant body measures through various sensitivity analyses and our conclusions remained consistent. Models showing consistency with our main findings included (1) additional adjustment for household income, diabetes status (ie, chronic diabetes and gestational diabetes), recruitment site, and EchoMRI measurement room temperature, (2) additional adjustment for maternal diet, and (3) additional adjustment for birth weight (Supplementary Table S2 [29]). Since the effect estimates of maternal stress on FM% changed more than 10% in the model with birthweight adjusted, we further conducted a mediation analysis to understand the indirect effect of birthweight. We observed that the direct effect of maternal stress during late pregnancy on FM% remained significant (beta estimate per 1-SD increase in PSS: primary analysis [total effect]—0.78; 95% CI, 0.13-1.44; *P* = .023; mediation analysis—0.63; 95% CI, 0.05-1.24; *P* = .04) while the indirect effect through birthweight was not statistically significant (Supplementary Table S3 [29]). We also observed similar results when we modeled maternal stress during the early pregnancy as exposure and assessed its effect on birthweight and 1-month infant body composition measures (Supplementary Table S4 [29]).

## Discussion

This study found that higher maternal perceived stress during late pregnancy is associated with higher fat mass related body composition measures in infants at 1 month, after adjusting for covariates. The association was most evident in male infants and in preterm infants compared the overall population, female infants, and term birth. The associations were similar across all the fat mass-related body composition measures. These results suggest that maternal perceived stress even at moderate levels contributes to an increase in the percentage of fat mass in infancy. Though the absolute percentage change at 1 month was small, the effect may be much larger when compounded across all of childhood. The results also suggest it may be important to devote greater effort to the management of stress levels in pregnancy.

Several epidemiological studies have examined prenatal stress and offspring adiposity during early childhood. These studies have used a wide range of adiposity measures, including but not limited to BMI without *z*-score, body weight, and body fat mass. Most of these studies have suggested that prenatal stress is associated with increased offspring adiposity [12, 16], which is consistent with the current study, although a few of them have reported results with the opposite direction [13, 14]. These mixed findings may reflect the fact that these studies measured different dimensions and timing of stress [34] and how it relates to offspring adiposity. We might not be able to compare the magnitude of effect of prenatal stress directly because most of the studies measured adiposity during different life stages (ie, infancy, childhood, or adolescence) and it may take more time for the effects of maternal stress on child obesity to accumulate (months or years) because of differences in weight-related parenting practices and maternal feeding. Future studies should further understand the clinical relevance of the observed increase in fat mass in relation to childhood obesity risk as there is limited clinical guideline regarding the topic during early infancy.

Importantly, stronger effects of maternal perceived stress on fat mass-related measures were observed among male infants. Research has shown that in response to challenging conditions, male fetuses tend to maintain or increase their growth rate, whereas female fetuses often slightly decrease their growth [16]. Various theories for this difference have been discussed, with 2 key explanations standing out. Evolutionarily, it has been suggested that in stressful intrauterine environments, males fetuses tend to accelerate their growth and prioritize physical development while females fetuses are thought to prioritize the preservation to improve fetal viability [35]. Physiologically, some studies have reported sex differences in placenta responsivity to stress and immune and hormonal changes during gestation [17]. For example, cortisol released during pregnancy has sex-specific effects on fetal behavior [36]. These mechanisms might provide a plausible explanation for the observed results, and future studies should further investigate how infant sex and maternal stress interact to impact fetal growth.

Furthermore, our study also found that preterm birth status modifies the influence of maternal perceived stress on infant adiposity at 1 month. Although the distribution of infant sex in preterm infants (45% female) appeared to be equal, which might suggest that preterm birth status may not modify this relationship via infant sex, and we might not be able to rule out the role of sex in the modification effect of preterm birth because of small sample size. Several studies have suggested that preterm infants tend to have higher percent-fat mass compared to term-born infants [37]. One hypothesis is that preterm-born infants might experience insufficient nutritional supply due to limited amino acid delivery to match the high fetal protein need, and therefore catch-up growth occurs to match insufficient protein intake [38]. This process may lead to a state of skeletal muscle insulin resistance, which seems to be strictly associated with an accumulation of fat mass rather than fat-free mass [39]. Maternal stress has also been associated directly with preterm birth [40] indicating a need for future investigation into how preterm birth and maternal stress interact to impact adiposity during early infancy with a larger sample size.

The results of the current study are notable for several reasons. First, the study population comprised an underrepresented group in the literature of predominately low-income Hispanic participants who traditionally have been excluded from previous research. Additionally, EchoMRI was utilized, which has shown high precision and reproducibility for measuring infant FM and LM [26]. In related literature, BMI is used to assess obesity status among children because of its measurement efficiency. However, BMI can only be considered a proxy for body fat proportion [41] and is not recommended for use in children younger than age 2 years [42]. When using BMI as a measure, it is possible to overestimate the effect of maternal stress in children who have high LM or children who have low body weight but a high proportion of FM. Therefore, the current study may provide a more accurate measure of body composition and adiposity compared to other measures such as weight and BMI. Last, this is one of the few studies to explore how infant sex and preterm birth status modify the association of maternal perceived stress on infant adiposity.

The current study has several limitations. First, we can only assess the perceive stress effect during late pregnancy, but the results have limited ability to represent the perceived stress effect for the whole third trimester as 1-time measured PSS can only reflect maternal stress for past 30 days. We were also unable to disentangle effects of stress at 1 month postpartum on infant obesity from effects of stress during late pregnancy. Mothers who are stressed during the first month may engage in different parenting behaviors (eg, feeding, pattern of putting infant to sleep) that could affect infant adiposity at 1 month outside of the effects of stress during late pregnancy [43, 44]. Although, some studies suggest that the third trimester is the most critical window for fetal body composition programming [8]. Another limitation is that the sample size of our study was relatively small, especially in the preterm-born infant group, potentially limiting power to detect true associations. Additionally, we do not yet have the ability to assess the effect of maternal perceived stress on infant body composition longitudinally due to limited sample sizes in older children to date. Future research should be conducted in a longitudinal fashion to understand the long-term effect of maternal perceived stress on infant body composition. Last, although several sensitivity analyses were performed to assess the robustness of our results, we acknowledge that residual confounding remains a possibility.

## Conclusion

In conclusion, our findings suggest that maternal perceived stress during late pregnancy may increase adiposity at early infancy, and this association is modified by infant sex and preterm birth status. Higher adiposity in childhood is associated with adult obesity and many other negative health consequences [3]. In this study, we have focused on PSS that measures maternal stress in general but in future research other stress measurements should be considered simultaneously to provide a more comprehensive picture of the effect of maternal stress. Moreover, although most of the women were considered to be in the low-stress category, the effects of their stress on their offspring adiposity were still notable. Interventions to reduce maternal stress should be further promoted, especially in populations at high risk of stress or obesity, and in populations that may have poor coping mechanisms or stigma surrounding mental health.

## Data Availability

Restrictions apply to the availability of all data generated or analyzed during this study because they were used under license. The corresponding author will on request detail the restrictions and any conditions under which access to some data may be provided.

## Abbreviations

BMI: body mass index
FM: fat mass
FMI: fat mass index
LM: lean mass
MADRES: Maternal and Developmental Risks from Environmental and Social Stressors
PSS: Perceived Stress Scale
